# Conserved microbiota among young *Heliconius* butterfly species

**DOI:** 10.7717/peerj.5502

**Published:** 2018-10-02

**Authors:** Bas van Schooten, Filipa Godoy-Vitorino, W. Owen McMillan, Riccardo Papa

**Affiliations:** 1 Department of Biology, University of Puerto Rico, Rio Piedras Campus, San Juan, Puerto Rico; 2 Department of Microbiology and Medical Zoology, School of Medicine, University of Puerto Rico, Medical Sciences Campus, San Juan, Puerto Rico; 3 Smithsonian Tropical Research Institute, Panamá, Panama; 4 University of Puerto Rico, Molecular Sciences and Research Center, San Juan, Puerto Rico

**Keywords:** Microbiota, Bacterial diversity, 16S rRNA, Butterflies, Spiroplasma, Lepidoptera, Chlamydiae, Evolution, 16S, Microbiome

## Abstract

**Background:**

Insects are the most diverse group of animals which have established intricate evolutionary interactions with bacteria. However, the importance of these interactions is still poorly understood. Few studies have focused on a closely related group of insect species, to test the similarities and differences between their microbiota. *Heliconius* butterflies are a charismatic recent insect radiation that evolved the unique ability to use pollen as a protein source, which affected life history traits and resulted in an elevated speciation rates. We hypothesize that different *Heliconius* butterflies sharing a similar trophic pollen niche, harbor a similar gut flora within species, population and sexes.

**Methods:**

To test our hypothesis, we characterized the microbiota of 38 adult male and female butterflies representing six species of *Heliconius* butterflies and 2 populations of the same species. We sequenced the V4 region of the 16S rRNA gene with the Roche 454 system and analyzed the data with standard tools for microbiome analysis.

**Results:**

Overall, we found a low microbial diversity with only 10 OTUs dominating across all individuals, mostly *Proteobacteria* and *Firmicutes,* which accounted for  99.5% of the bacterial reads. When rare reads were considered, we identified a total of 406 OTUs across our samples. We identified reads within Phyla *Chlamydiae*, found in 5 butterflies of four species. Interestingly, only three OTUs were shared among all 38 individuals (*Bacillus, Enterococcus* and *Enterobacteriaceae*). Altogether, the high individual variation overshadowed species and sex differences. Thus, bacterial communities were not structured randomly with 13% of beta-diversity explained by species, and 40 rare OTUs being significantly different across species. Finally, 13 OTUs, including the intercellular symbiont *Spiroplasma,* varied significantly in relative abundance between males and females.

**Discussion:**

The* Heliconius* microbial communities in these 38 individuals show a low diversity with few differences in the rare microbes between females, males, species or populations. Indeed, *Heliconius* butterflies, similarly to other insects, are dominated by few OTUs, mainly from Proteobacteria and Firmicutes. The overall low microbial diversity observed contrasts with the high intra-species variation in microbiome composition. This could indicate that much of the microbiome maybe acquired from their surroundings. The significant differences between species and sexes were restricted to rare taxa, which could be important for microbial community stability under changing conditions as seen in other host-microbiome systems. The presence of symbionts like *Spiroplasma* or *Chlamydiae*, identified in this study for the first time in *Heliconius*, could play a vital role in their behavior and evolution by vertical transmission. Altogether, our study represents a step forward into the description of the microbial diversity in a charismatic group of closely related butterflies.

## Introduction

Insects have been co-evolving with their bacterial symbionts for at least 250 million years, developing fascinating complex interactions (Wernegreen, 2002; Toft & Andersson, 2010; Brucker & Bordenstein, 2012). A growing appreciation for these relationships is a result of the realization of the great impact that microbiota can have on the physiology, health, reproduction, behavior, growth, thermal tolerance and longevity of the host (Cox-Foster et al., 2007; Russell et al., 2009; Vásquez et al., 2012). Nonetheless, we still lack a fundamental understanding of the microbial communities associated with insects, the degree of host specialization, and the overall functional importance of these communities to the ecology of their hosts.

*Heliconius* is one of the best known modern insect radiations. This insect genus is composed of over 40 species and hundreds of geographic population variants. The radiation is characterized by the evolution of several lineage-specific innovations including an ecological shift to pollen feeding. *Heliconius* is unique among butterflies in their ability to collect and utilize pollen as a protein source (Harpel et al., 2015). The transition to pollen feeding is hypothesized to be important in the butterflies’ ability to synthesize toxic compounds and to enable a very long adult life (Gilbert, 1972). Pollen feeding is also associated with a rapid increase in brain size (Sivinski, 1989; Montgomery, Merrill & Ott, 2016) and the evolution of a suite of complex behaviors. Some of these traits include trap-line feeding, gregarious roosting, and elaborate mating strategies (Brown Jr, 1981). Moreover, pollen feeding can strongly shape the gut microbiota, the metabolism and various physiological traits, which affects the reproductive biology and life history traits of insects (Gilbert, 1972; Engel, Martinson & Moran, 2012). Recent studies suggest the role of bee gut bacteria in the metabolism of pollen and pollen-derived substrates, including flavonoids and outer pollen wall components, attributed to Lactobacilli and Bifidobacteria (Kešnerová et al., 2017). Interestingly, Lactobacilli have been suggested to prevent spoilage of the pollen that wild bees provision for their young by inhibiting the growth of fungi (McFrederick, Vuong & Rothman, 2018).

In recent times a study on ants from the same colonies, species, genera and tribes sharing trophic niches have shown similarities between their microbial communities (Anderson et al., 2012). Our study wanted to test if different pollen-feeding *Heliconius* butterflies species, sexes and populations present a similar microbiota due to a common feeding strategy. To date, there has only been a previous survey of microbial diversity in *Heliconius* (Hammer, McMillan & Fierer, 2014), which focused on the microbial similarities and differences between caterpillars and adults of *H. erato*. Although this study identified several bacterial OTUs shared between larvae and adults, it also found a profound turnover in the bacterial communities between life stages. While adult *H. erato* didn’t show any sexual differences in microbial composition, wild individuals were dominated by *Proteobacteria* (74%), followed by *Firmicutes* (13%), *Bacteroidetes* (9%), Tenericutes (3%). This result roughly agrees with results reported by Yun et al. (2014), which characterized the gut microbial community across 218 species of insects representing 21 orders.

We hypothesize that *Heliconius* butterflies sharing the same niches and diet may a similar microbiota. With our work we characterized the bacterial community in a diverse group of adult butterflies with our work we characterized the bacterial community on six common Heliconius species found in forests around Gamboa, Panama, to test how these communities varied among individuals, species, populations and sexes. These diverse group of adult butterflies of the same genus included both closely related species, and species that diverged early in the radiation, ∼12–15 million years ago (Kozak et al., 2015). Considering the diverse ways in which bacteria can influence insect evolution, differences between species could be intertwined with speciation and/or host-plant adaptation. Similarly, differences between male and female butterflies could be caused by bacteria induced pheromones or other sex specific chemical compounds produced by specific microbial communities as in locusts and *Drosophila* (Dillon, Vennard & Charnley, 2002; Sharon et al., 2013; Leftwich et al., 2017).

## Material and Methods

### *Heliconius* butterfly collection

**Figure 1:**
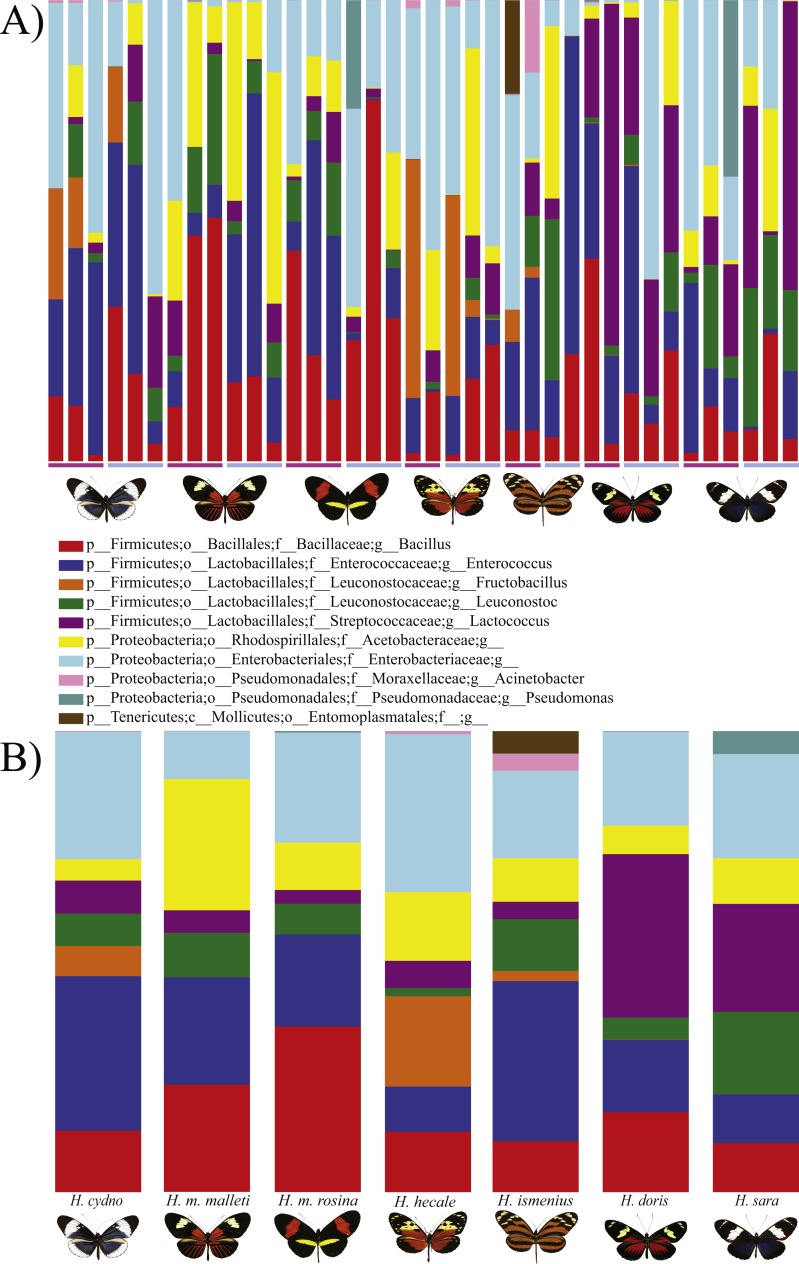
Distribution of Bacterial OTUs across individuals (A) and species (B). The plot shows the percentage of total reads mapped to specific OTUs. Only the 11 OTUs present in more than 0.5% of the reads are shown, where p, phylum; c, class; o, order; f, family; g, genus. From left to right: *H . melpomene malleti, H. melpomene rosina, H. cydno chioneus, H. hecale, H. ismenius, H. sara* and *H. doris*. Within species (A), female and male butterflies a grouped together (pink bar and blue bars, respectively). (B) The averages per species after rarefication to 552 reads.

**Figure 2:**
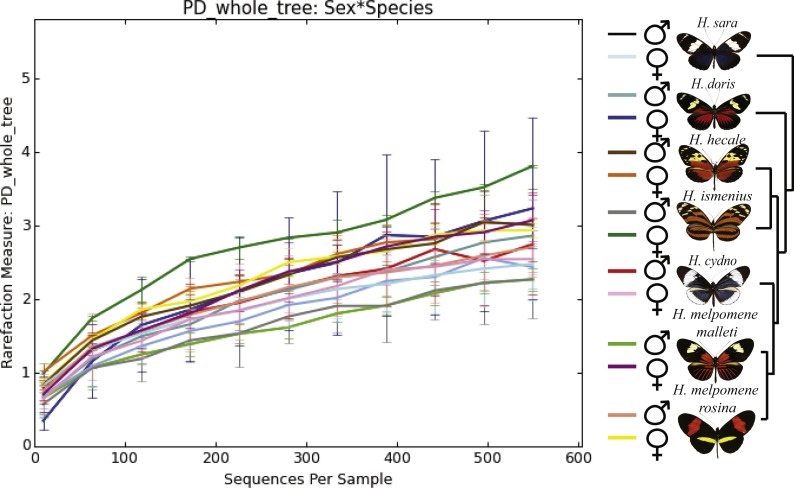
Alpha phylogenetic diversity measures do not differ significantly between species or sexes. Rarefaction was done to 552 to be able to include the butterfly with the least reads.

**Figure 3:**
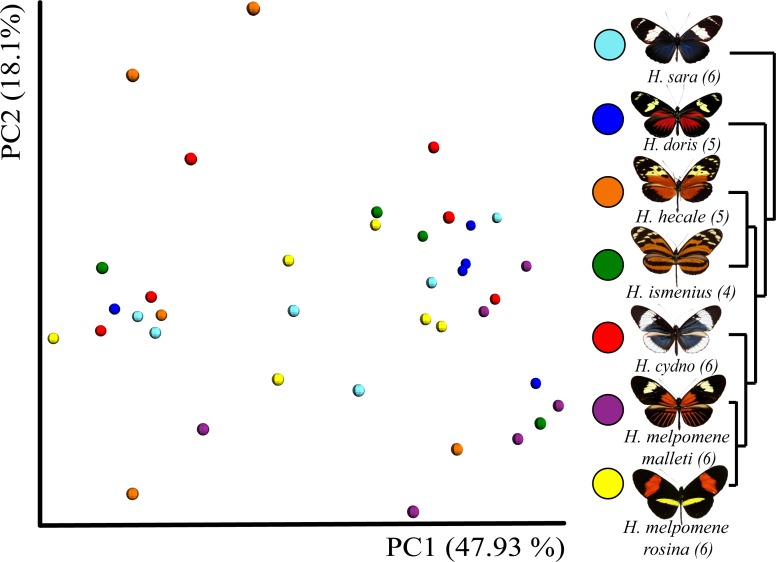
Beta diversity comparisons by Principal Coordinate Analyses (weighted Unifrac) indicates a slight, yet significant, separation according to species. Species explain 13% of variation in beta diversity (ANOSIM *R* = 0.1321, *P* = 0.029, perm = 999) and PERMANOVA testing Pseudo-F: 1.50, indicated significant differences in the bacterial structure according to species, *p*-value = 0.001). We found no significant difference between sexes (ANOSIM *R* =  − 0.0090, *P* = 0.499, perm = 999). Rarefaction was done to 552 to be able to include the butterfly with the least reads.**** Number of butterflies per species is shown in brackets next to the species name. Phylogenetic tree shows relationship between species.

Our experimental approach, done in Gamboa, Panama in 2011 (9°07′11.8″N 79°42′05.6″W), the same location as Hammer, McMillan & Fierer (2014), was to rear and house caterpillars and adult butterflies under controlled experimental conditions in outside insectaries on their natural host plants. Captive rearing had an effect on the host microbial community in adult *H. erato,* presumably due to differences in the types of host plants available (Hammer, McMillan & Fierer, 2014). However, standardized conditions allowed us to somewhat control environmental variation and better understand any species-specific and/or sex-specific differences in the bacterial communities. Of every (sub) species three males and three females were collected (except *H. ismenius* which had two individuals of both sexes). Host plants were reared next to the rainforest where the butterflies naturally occur. After emerging, the adult butterflies lived for 2 days and were fed using plants they also feed on in the rainforest, and supplemental sugar water—a common dietary supplement used in butterfly gardens. These plants are also visited by wild butterflies and other insects when stored outside the cage. The butterfly species of our study are shown in: *Heliconius cydno chioneus, H. melpomene malleti* (not native to Panama), *H. melpomene rosina, H. hecale melicerta, H. ismenius boulleti, H. doris viridis* (red form) and *H. sara magdalena* (Fig. 1). For their phylogenetic relationships see phylogeny present Figs. 2 and 3, which is based on (Kozak et al., 2015).

### Dissection and DNA isolation

Butterflies were collected and stored in RNAlater at −20 °C. The last 3 segments of the abdomen were sterilized on the outside with 70% ethanol and cut from the rest of the body. The end of the abdomen than was sonicated (50 to 60 Hz, 115 V, 10% A; Branson Ultrasonics, 10 s) after suspending it in 600 ml TE buffer. After the sonication, samples were centrifuged (6,000 rpm) for 5s to let large chitin particles sink to the bottom. 500 mL of the fluid was then transplanted to a new 1.5 ml tube and centrifuged (7,500 rpm 10 min). We removed as much fluid as possible and the pellet was re-suspended in ATL buffer (supplied in the DNeasy kit; Qiagen, Valencia, CA, USA). The remainder of the genomic DNA extraction was done according to the DNeasy kit manufacturer’s protocol. The only modification of the protocol was that the gut was lysed overnight by using only 25 µl of AE buffer.

### 16S rRNA V4 amplification and 454 sequencing

The DNA was amplified using PCR with a BIO RAD MyCycler thermocycler. The thermal cycling conditions were an initial 3-min denaturation step at 94 °C, 35 cycles at 94 °C for 30 s, 50 °C for 30 s, and 72 °C for 60 s, with a final 10-min extension at 72 °C. No hot start was used. We targeted the V4 region of the 16S SSU rRNA, using primers 515F 5′-GAGTGCCAGCMGCCGCGGTAA-3′and 806R 5′-CCGGACTACHVGGGTWTCTA AT-3′. Although we had negative controls for PCR, these were not sequenced. Samples were barcoded and multiplexed on the Roche 454 using the 16S Amplicon rRNA protocol.

### Bioinformatic data analyses

Data was analyzed using QIIME 1.9 (Caporaso et al., 2010). Split libraries were used with minimum sequence length of 240 and a minimum quality score of 30 and a maximum number of primer mismatches of one. Operational taxonomic units were defined at the standard 97% sequence divergence. Singletons, Chloroplast and unassigned reads were filtered from the alignment. All other analyses were done using default parameters incl. chimera detection in QIIME (Caporaso et al., 2010).

Diversity analyses (alpha and beta-diversity) were done using a rarefaction level of 552 reads so the sample with the least sequences could be included. We also looked at alpha- and beta-diversity results with rarefaction at 4,825 excluding a *H. doris* male and a *H. m. rosina* male and found results to be very similar. Rarefaction at 500 read depth has been shown to be sufficient to detect biological differences in bacterial communities of insects (Jones, Sanchez & Fierer, 2013; Hammer, McMillan & Fierer, 2014). This random subsampling is also useful to mitigate biases due to differences in sampling depth. The alpha and beta-diversity analysis were done with QIIME script core_diversity_analyses.py with default values. Two Sample *t*-tests via monte-carlo permutations were used for alpha-diversity (Phylogentic Distance) differences between sexes and species. The QIIME script compare_categories.py was used to see if species and sexes differed in beta-diversity, with the ANOSIM method based on the weighted-unifrac distance-matrix. Sexes were characterized per species and together for all species. For each group comparison (species and sex), significance tests were computed including the maximum likelihood statistical significance tests that determine whether OTU presence/absence is associated with a category in the metadata. The goodness-of-fit or log-likelihood ratio parametric test (*G*-test) compares the ratio of observed OTU frequencies in the sample groups to the expected frequencies based on the null hypothesis (all sample groups have equal OTU frequencies). The heatmap was generated with heatmap.3 function in R (Zhao et al., 2014). Data normalization was done through DESeq2 negative binomial Wald normalization for visualization purposes. This normalization step was implemented in QIIME using the script normalize_table.py. To verify which genus the OTU that was placed in family Entomoplasmatales most likely belonged to, the sequence was used to BLAST (Altschul et al., 1990) against the Greengenes database (McDonald et al., 2012) to find which known bacteria showed the highest sequence similarity.

## Results

### Individual microbiota comparisons

The Roche 454 amplicon run of 38 *Heliconius* butterfly samples resulted in 295,087 good-quality reads with an average of 7,765 reads (lowest 552 reads, highest 13,744 reads) per sample, with a 252 bp read length (Table S1). The whole 406 OTUs were distributed across samples from a minimum of 29 OTUs to 186 OTUs per sample (Table S1). We found a total of 7 bacterial phyla. The reads were dominated by Firmicutes (∼62% of reads) and Proteobacterial OTUs (∼37% of reads) (Table S2), with the rest of the OTUs occupying <1% of the reads distributed between Actinobacteria, Bacteroidetes, Chlamydiae, Planctomycetes, and Tenericutes.

In general, OTU similarity among individuals was high with 10 OTUs accounting for 92% of the reads across samples (Fig. 1). Nonetheless, only 3 OTUs, *Bacillus, Enterococcus* and *Enterobacteriaceae,* were present in all 38 samples and none were restricted to all individuals of a single species or sex (Fig. S1). If we exclude the 10 most abundant OTUs, the remaining 395 OTUs found represent ∼8% of total reads (Table 1). Lactic acid bacteria (*Lactobacillales* groups such as *Lactobacillus*, *Fructobacillus* and *Enterococcus*) were prevalent among all samples. These mutualistic bacteria are likely to produce by-products to promote growth of core microbial members. Finally, our data highlights a wide inter-individual variation in abundance between the 38 samples with phyla ranging from 98.6% Proteobacteria and 1.1% Firmicutes in a *H. doris* male to 23.2% Proteobacteria and 76.7% Firmicutes in a *H. hecale* female (Fig. 1). This high individual variation is also reflected to some degree in the beta-diversity plot (Fig. 3) by the total lack of clustering per species or sex.

**Table 1:** Percentage of raw reads of the 11 most common OTUs, averaged per (sub)species.

Phylum **or Order**	**Family**	**Genus**	***H. cydno*n=6**	***H. m. malleti****n* = 6	***H. m. rosina****n* = 6	***H. hecale****n* = 5	***H. ismenius****n* = 4	***H. doris****n* = 5	***H. sara****n* = 6
**Firmicutes**									
Bacillales	Bacillaceae	Bacillus	13.3%	23.3%	35.9%	13.1%	11.0%	17.4%	10.7%
Lactobacillales	Enterococcaceae	Enterococcus	33.5%	23.3%	20.0%	9.8%	34.7%	15.7%	10.6%
	Leuconostocaceae	Fructobacillus	6.6%	0.0%	0.0%	19.6%	2.2%	0.1%	0.0%
		Leuconostoc	7.0%	9.6%	6.7%	1.8%	11.2%	4.7%	17.8%
	Streptococcaceae	Lactococcus	7.2%	4.9%	3.0%	5.9%	3.8%	35.4%	23.4%
**Proteobacteria**									
Rhodospirillales	Acetobacteraceae	g__	4.6%	28.5%	10.3%	14.9%	9.5%	6.2%	9.9%
Enterobacteriales	Enterobacteriaceae	g__	27.6%	10.4%	23.9%	34.3%	19.0%	20.4%	22.6%
Pseudomonadales	Moraxellaceae	Acinetobacter	0.2%	0%	0%	0.6%	3.7%	0%	0%
	Pseudomonadaceae	Pseudomonas	0%	0%	0.3%	0%	0%	0.1%	4.9%
**Tenericutes**									
Entomoplasmatales	f__	g__	0%	0%	0%	0%	4.8%	0%	0%

### Microbiota comparisons among Species

**Figure 4:**
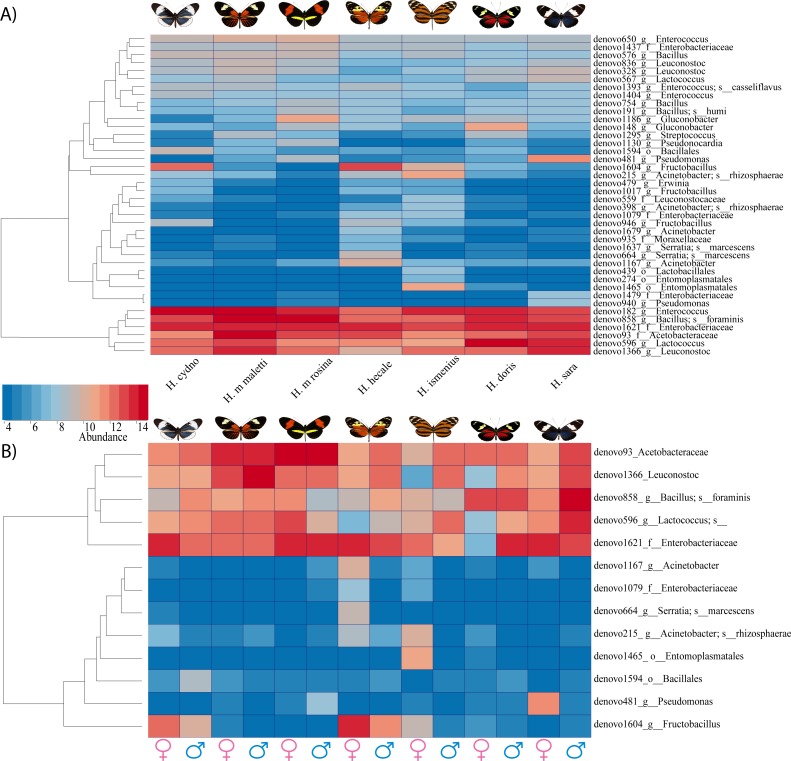
Bacterial significant differences between species, subspecies and sexes. (A) A heatmap of reads found per butterfly of the 40 OTUs which differed in abundance between species based on *G*-test with Bonferroni corrected *p*-values. (B) Plot abundance of the 13 bacteria found to differ between males compared to females (all species pooled)

While the conserved microbiome between the six sequenced species consists of only three shared OTUs, a closer look at the core microbiome within species revealed a highest core microbiome of 11 OTUs in *H. doris* followed by *H. sara* with 7 OTUs (Fig.S2). Alpha-diversity did not show significant differences in number of OTUs between *Heliconius* species (Fig. 2, Table S3). Nonetheless, even with inter-individual variation, bacterial diversity was not distributed randomly among species. Indeed, community structure was evaluated as a beta-diversity measure between species, considering the abundance and composition of microbes. Using a weighted Unifrac distance-matrix, we determined that butterfly species explained 13% of the variation in beta-diversity (Fig. 3 ANOSIM *R* = 0.1321, *P* = 0.029, perm = 999), despite the dominant 10 OTUs. In fact, PERMANOVA testing Pseudo-F: 1.50, *DF* = 1.51, indicated significant differences in the bacterial structure according to species, (*p*-value = 0.001). Although beta-diversity doesn’t tell which bacteria cause the difference, a log-likelihood ratio parametric test we revealed 40 OTUs that changed significantly in abundance between species (Fig. 4A). Certain groups of microorganisms such as Lactobacillales and *Pseudonocardia* showed distinct abundance differences between species. While Lactobacillales OTUs were less abundant in *H*. *doris* and *H*. *ismenius*, *Pseudonocardia* OTUs were found more abundant in *H*. *doris* and *H*. *m*. *rosina* (Fig. S3). We also found that *H. hecale*, *H. cydno* and *H. ismenius* share unique taxa that are not shared with other butterflies (including *Erwinia*). Finally, some OTUs were more abundant in some species: *Bacillus* in *H. m. rosina* and *H. m. malleti,* Enterobacteriaceae in *H. cydno*, Entomoplasmatales and *Acinetobacter* in *H. ismenius*, *Fructobacillus* in *H. hecale*, *Lactococcus* in *H. doris*, and *Pseudomonas* in *H. sara* (Figs. 1A, 1B, Fig. S3).

### Microbiota comparisons between populations

We included two distinct populations of *Heliconius melpomene*, *H. m. malleti* and *H. m. rosina,* to see if differences arise at the subspecies level, and if generally differences between species are greater than between population. In our analysis of alpha-diversity (Fig. 2), beta-diversity (Fig. 3) and core microbiome (Fig. S2), they differ as much as from each other as from *H. cydno,* their close relative. However, changes in relative abundance of specific microorganisms were found between the two populations of *H. melpomene*. These slight differences include the fact that *H. m. malleti* have more *Acetobacteraceae* and less *Bacillus* and Enterobacteriaceae than *H. m. rosina* (Fig. 2).

### Microbiota comparison between sexes

Alpha-diversity across gender for all *Heliconius* species showed no significant differences between sexes (Fig. 2, Table S3). Sexes also showed no difference in beta-diversity (ANOSIM *R* =  − 0.0090, *P* = 0.499, perm = 999). Despite those results, the abundance of 13 OTUs changed significantly (*p* < 0.05) between males and females (Fig. 4B). While female butterflies presented more *Fructobacillus*, *Enterobacteriaceae* and *Entomoplasmatales,* males showed more *Acetobacteraceae* and *Lactococcus*.

### Entomoplasmatales

Using BLAST, we found that the OTU previously classified as a member of the family Entomoplasmatales is most likely a *Spiroplasma*. The first four hits were four strains of *Spiroplasma sp*. with the same Score = 397 bits (250); Expect = e−110; Identities = 251/252 (99%). These results indicate that *Spiroplasma sp.* was found in one *H. doris* female and three *H. ismenius,* two females and one male, of which one female had 19% of her reads belonging to this bacterium (Fig. 1A)*.*

## Discussion

Our study revealed that the *Heliconius* adult butterfly microbial richness is relatively low, compared to herbivorous insects feeding in recalcitrant food components and mammals (Yun et al., 2014). This is exemplified by at the overall number of OTUs, which ranged from 29 to 186 OTUs, and from the presence of only 10 dominant OTUs across the whole microbiome of the guts of our 38 individuals. However, the low diversity found in our study is in line with a recent study on *H. erato,* where an average of 43 OTUs were found of which 12 OTUs have been found to dominate the microbiome (abundance >1%) in nine wild individuals (Hammer, McMillan & Fierer, 2014). Interestingly, the low microbiome complexity doesn’t seem a unique characteristic of *Heliconius* butterflies. The same simple microbiota composition was also found in *Spodoptera littoralis* insects (Chen et al., 2016) as well as in different species of mosquitoes from Kenya (Osei-Poku et al., 2012). Our results are thus generally in agreement with the current microbiome literature of insects. As a matter of fact, gut bacterial species richness in insects is considered to be low, except for wood eating insects such as termites (Colman, Toolson & Takacs-Vesbach, 2012; Yun et al., 2014). Our study focused only on the gut microbiota of the butterflies and no data have been collected from the plants they feed or the sugar water that was utilized as food supplement. Our study was designed to provide the first characterization of the microbiome in a group of closely related butterfly species. Thus, due the lack of data from food source we cannot determine the effect of pollen feeding and sugar water on the observed conserved microbiota.

The two phyla, Proteobacteria and Firmicutes, that dominate the microbiome in our study are the same dominant ones found in two recent insect microbial survey studies from (Hammer, McMillan & Fierer (2014) and Yun et al. (2014). A closer look at the overall microbial diversity highlight a total of seven phyla in our study compared to the 13 phyla in the Lepidoptera by Yun et al. (2014). However, we identified for the first time the presence of one rare OTU belonging to the phyla Chlamydiae, which was found in five butterflies representing four species, but was absent in the study of Yun et al. (2014) and has not been reported in Lepidoptera before. This finding could be very important due to the fact members of the Chlamydiae are known to be symbionts or pathogens in insects and other Eukaryotes (Moran, McCutcheon & Nakabachi, 2008). Thus, future studies on the effect of the presence of Chlamydiae in butterflies could reveal important insights into the interaction of this bacteria with the physiology or life history of these insects.

When we compare our study to the only other work published on *Heliconius* butterflies by Hammer, McMillan & Fierer (2014), we found a strong overlap between the two datasets. The only discrepancy is represented by the much higher presence of Bacteroidetes (9%) in Hammer, McMillan & Fierer (2014) compared to our data (0.02%). One possible explanation of this difference could be that while Bacteroidetes are almost nonexistent in our recently eclosed butterflies (2 days old) they become abundant after few days of pupal emergence represented by the 4-day-old butterflies studied by Hammer, McMillan & Fierer (2014). In concordance with the results by Hammer, McMillan & Fierer (2014) this may indicate that metamorphosing insects increase their microbiota diversity over time, after eclosing, as gut walls become a more stable surface for microbial colonization.

Despite 10 dominant OTUs occupying all species in this study, only three were found present in all butterflies, suggesting the microbiome is conserved among species and has a low diversity. Despite this dominant trend, we found that butterflies species explined 13% of beta-diversity, with 40 low abundant OTUs differing significantly between species. We also found that the two population of *Heliconius melpomene* (*H. m rosina* and *H. m. malleti)* separate slightly in beta-diversity analyses (Fig. 3). The observation that differences between *Heliconius* species were restricted to rare OTUs can be rather important since rare taxa have been shown to be important in other biological systems. Distinct studies in soils (Hausmann et al., 2016), human skin, human gut and the coral holobiont (Shade et al., 2014; Godoy-Vitorino et al., 2017) have suggested that phylotypes with very low abundance (rare taxa) are likely to be functionally relevant.

The few differences in beta-diversity suggest that the dominant microbiota is conserved between sexes. Sex differences are restricted to only 13 rare OTUs, which showed significant differences in abundance between males and females (*p* < 0.05). These rare bacteria represent the only differences in microbial patterns associated with butterfly’s sex, which could represent, as mentioned above, candidate rare taxa with likely important functions. Indeed, it is possible that these rare microorganisms are important for responding to changes in environmental conditions, and act as a reservoir that help to stabilize and buffer the dominant microbial community (Shade et al., 2014; Jousset et al., 2017).

When comparing abundances between species and sexes, one should also consider individual microbial variation. In our study, we found a large variation between individuals, as shown by the 11 butterflies dominated by Proteobacteria while another 25 butterflies were dominated by Firmicutes. Similar pattern of variation at the individual level was also observed by Hammer, McMillan & Fierer (2014), and in other studies where intraspecific variability in microbial composition has also been associated with diet- and gut-associated microbiota (Priya et al., 2012; Staudacher et al., 2016).

Diverse aspects can influence the microbiome composition and abundance of specific groups between the different *Heliconius* butterflies. Dietary factors may represent one of the main elements that modulate the gut microbiota of butterflies, as already demonstrated in other organisms (Colman, Toolson & Takacs-Vesbach, 2012). However, *Heliconius* areunique among butterflies due the fact they feed on pollen. Their simple diet (sugar and protein) can partially explain their low gut diversity. Moreover, the flowers could function as breeding and sharing grounds of microorganisms for the insects that will visit them, as supported by the broad similarities among pollinating insects (Ushio et al., 2015). This interaction can homogenize the microorganisms’ intake associated to food, especially in groups of individuals that inhabit the same location and thus visit the same flowers. Possibly such interaction could also extend to birds and insects’ pollinators (Vannette & Fukami, 2017).

*Heliconius* butterflies exhibit complex behavior such as host-plant choice and oviposition, which can explain difference between species (Supple et al., 2014). For example, *H. sara* and *H. doris* are two species that display gregarious oviposition behavior, resulting in larvae growing near each other, usually on a single leaf (Beltrán et al., 2007). All the above life history traits can play a role in the physicochemical characteristics in the gut compartments of different butterfly species (whether being pH, O_2_ levels or substrate availability), which can influence their microbiome variation.

From our data, Lactobacillales, emerged as some of the few taxa that significantly changed in abundance between species. This group of microorganisms have previously been identified in the bee gut microbiota and associated with the metabolism of pollen and pollen-derived substrates, including flavonoids (Kešnerová et al., 2017). Moreover, Lactobacillales seem to provide anti-fungal effects in pollen provisions (McFrederick, Vuong & Rothman, 2018) and play a key role in the bee health and sociability (Rangberg et al., 2015). These lactic acid bacteria have also found to be dominant members of the microbiota of other insects, including some species of Hymenoptera (McFrederick et al., 2013). Among the other important functions suggested for *Lactobacilli*, the effect on the regeneration of the gut’s epithelia (Jones et al., 2013), and the possible influence in mating preferences by changing the levels of cuticular hydrocarbon sex pheromones (Matos & Leulier, 2014) have been reported. However, the exact mechanisms with which the *Lactobacilli* achieve this, remain to be elucidated (Sharon et al., 2010). Although the data in the current study cannot provide direct evidence of the functional capabilities of Lactobacillales, they are likely to have similar functions such as pollen metabolism and gut health in *Heliconius* butterflies.

Finally, one of the most intriguing results emerged from our data is the presence of an intra-cellular bacterium from the phylum Tenericutes, *Spiroplasma sp*. This is the first time that this facultative symbiont is described in *Heliconius.* Neither Muñoz et al. (2010) in 307 wild-caught *H. erato chestertonii* individuals, nor Hammer, McMillan & Fierer (2014) with 63 *H. erato* individuals, report this symbiont. However, we found that the raw data from Hammer, McMillan & Fierer (2014) had sequences belonging to the class Mollicutes to which *Spiroplasma* belongs. We believe that these sequences most likely are *Spiroplasma sp.,* although the classification is less certain due to the 151 bp sequence length compared to our 252 bp*.* More precisely, we found that the wild caught *H. erato* male WM5 from Hammer, McMillan & Fierer (2014) presented 33% of its reads homologues to *Spiroplasma*. It is known that *Spiroplasma* can infect insects in general, and also Nymphalidae, and is known to kill males in *Danaus chrysippus* (Jiggins et al., 2000). It is also known that *Spiroplasma* can enhance survival, as in *Drosophila hydei* by protecting from attack by parasitic wasps (Xie, Vilchez & Mateos, 2010). If and how these symbionts interfere with the *Heliconius* life cycle or behavior remains to be investigated.

## Conclusions

We found that the *Heliconius* microbial community, similar to other pollinator insects, is characterized by a low species richness, dominated mainly by Proteobacteria and Firmicutes (Hammer, McMillan & Fierer, 2014; Yun et al., 2014). This low diverse microbiota contrast with a high intra-species variation in microbiome composition. These two results seem to lead to the conclusion that the microbial composition of each individual recapitulate the distinct interaction that each butterfly might have with the surrounding environment. Nonetheless, our data show significant differences between species (13% of beta-diversity, and 40 OTUs) and sexes (13 OTUs). These differences emerged from the low abundant (rare) microbial taxa, suggesting that a deeper sequencing strategy with an Illumina approach might allow to resolve these differences with more confidence and power. We believe that the rare microbiota could actually be very important for the microbial community stability under changing conditions as seen in other host-microbiome systems and should deserve a future investigation. Finally, the presence of the intracellular symbionts like *Spiroplasma* or *Chlamydiae*, should be the focus of a functional study to better understand the effect of this bacteria in the butterfly’s behavior, life history and evolution. Altogether, our study represents a step forward into the description of the microbial diversity in a very charismatic group of butterflies not yet very well studied.

##  Supplemental Information

10.7717/peerj.5502/supp-1 Table S1 The biom file of the 38 butterflies

10.7717/peerj.5502/supp-2 Table S2 Number of reads and OTUs per butterfly species

10.7717/peerj.5502/supp-3 Table S3 *T*-test comparisons between phylogenetic diversity between female and male butterflies

10.7717/peerj.5502/supp-4 Figure S1 The abundance of the three OTUs present in all samples Shown is their relative abundance to each other averaged over the different (sub)species.

10.7717/peerj.5502/supp-5 Figure S2 The abundance of OTUs present in all samples for each of the 6 species and the two *H. melpomene* races Shown is their relative abundance to each other per butterfly.

10.7717/peerj.5502/supp-6 Figure S3 The abundance of 4 microbial genera that showed significant abundance differences between species

## Additional Information and Declarations

### Data Availability

The following information was supplied regarding data availability: Data was deposited at NCBI under BioSample accessions: SAMN07498379– SAMN07498418.
