# Supplementary material for: Conserved microbiota among young Heliconius butterfly species
Source: PeerJ. 2018 Oct 2;6:e5502. doi: 10.7717/peerj.5502 (PMC6173163; doi:10.7717/peerj.5502)
Supplement: Table S3 [file peerj-06-5502-s003.pdf]

Supplementary table 3. T-test comparisons between phylogenetic diversity between female and male butterflies

Group1	Group2	Group1 mean	Group1 std	Group2 mean	Group2 std	t stat	p-value
Maledoris	Malemelpomene_rosina	6.799	0.970	6.032	0.655	0.655	1
Maleismenius_boulleti	Femalemelpomene_malleti	5.933	1.177	7.411	0.644	-1.399	1
Malesara	Malecydno	6.072	1.190	5.411	0.092	0.607	1
Femaledoris	Femalehecale_melicerta	7.224	2.240	5.815	0.105	0.629	1
Malemelpomene_malleti	Femalemelpomene_malleti	5.398	0.588	7.411	0.644	-3.266	1
Maledoris	Femalemelpomene_rosina	6.799	0.970	6.741	0.399	0.072	1
Malemelpomene_malleti	Malecydno	5.398	0.588	5.411	0.092	-0.025	1
Malesara	Malemelpomene_malleti	6.072	1.190	5.398	0.588	0.719	1
Malemelpomene_rosina	Femalemelpomene_rosina	6.032	0.655	6.741	0.399	-1.164	1
Femaleismenius_boulleti	Malemelpomene_rosina	7.464	0.099	6.032	0.655	2.162	1
Malemelpomene_malleti	Femalemelpomene_rosina	5.398	0.588	6.741	0.399	-2.673	1
Malemelpomene_rosina	Malecydno	6.032	0.655	5.411	0.092	0.940	1
Malemelpomene_rosina	Femalemelpomene_malleti	6.032	0.655	7.411	0.644	-1.804	1
Femaleismenius_boulleti	Malecydno	7.464	0.099	5.411	0.092	15.186	1
Maleismenius_boulleti	Malemelpomene_rosina	5.933	1.177	6.032	0.655	-0.073	1
Femalehecale_melicerta	Femalesara	5.815	0.105	5.559	1.024	0.272	1
Maledoris	Malesara	6.799	0.970	6.072	1.190	0.557	1
Femaledoris	Femalesara	7.224	2.240	5.559	1.024	0.870	1
Malemelpomene_malleti	Malemelpomene_rosina	5.398	0.588	6.032	0.655	-0.875	1
Maleismenius_boulleti	Malemelpomene_malleti	5.933	1.177	5.398	0.588	0.521	1
Maleismenius_boulleti	Malehecale_melicerta	5.933	1.177	7.125	1.492	-0.736	1
Femalecydno	Malemelpomene_malleti	5.830	1.923	5.398	0.588	0.282	1
Femalehecale_melicerta	Malemelpomene_rosina	5.815	0.105	6.032	0.655	-0.328	1
Femaledoris	Femalecydno	7.224	2.240	5.830	1.923	0.472	1
Femalesara	Femalemelpomene_rosina	5.559	1.024	6.741	0.399	-1.520	1
Maledoris	Malemelpomene_malleti	6.799	0.970	5.398	0.588	1.556	1
Malehecale_melicerta	Femalesara	7.125	1.492	5.559	1.024	1.223	1
Maledoris	Femalesara	6.799	0.970	5.559	1.024	1.049	1
Femaledoris	Maleismenius_boulleti	7.224	2.240	5.933	1.177	0.510	1
Femalecydno	Maledoris	5.830	1.923	6.799	0.970	-0.450	1
Femalecydno	Femalemelpomene_malleti	5.830	1.923	7.411	0.644	-1.020	1
Femaledoris	Malemelpomene_rosina	7.224	2.240	6.032	0.655	0.511	1
Malesara	Malemelpomene_rosina	6.072	1.190	6.032	0.655	0.034	1
Malemelpomene_malleti	Malehecale_melicerta	5.398	0.588	7.125	1.492	-1.523	1
Femaleismenius_boulleti	Malemelpomene_malleti	7.464	0.099	5.398	0.588	3.815	1
Maledoris	Femalemelpomene_malleti	6.799	0.970	7.411	0.644	-0.656	1
Malesara	Malehecale_melicerta	6.072	1.190	7.125	1.492	-0.780	1
Femaledoris	Malemelpomene_malleti	7.224	2.240	5.398	0.588	1.042	1
Maleismenius_boulleti	Femalemelpomene_rosina	5.933	1.177	6.741	0.399	-0.850	1
Femalecydno	Malecydno	5.830	1.923	5.411	0.092	0.218	1
Malehecale_melicerta	Malemelpomene_rosina	7.125	1.492	6.032	0.655	0.755	1

Maleismenius_boulleti	Malecydno	5.933	1.177	5.411	0.092	0.443	1
Femalesara	Malemelpomene_rosina	5.559	1.024	6.032	0.655	-0.448	1
Maledoris	Femaleismenius_boulleti	6.799	0.970	7.464	0.099	-0.681	1
Femaleismenius_boulleti	Femalehecale_melicerta	7.464	0.099	5.815	0.105	11.422	1
Femalecydno	Femaleismenius_boulleti	5.830	1.923	7.464	0.099	-0.849	1
Femalehecale_melicerta	Femalemelpomene_rosina	5.815	0.105	6.741	0.399	-2.484	1
Femaledoris	Malehecale_melicerta	7.224	2.240	7.125	1.492	0.046	1
Femaledoris	Femalemelpomene_rosina	7.224	2.240	6.741	0.399	0.283	1
Femaleismenius_boulleti	Malehecale_melicerta	7.464	0.099	7.125	1.492	0.249	1
Femaledoris	Malesara	7.224	2.240	6.072	1.190	0.578	1
Malesara	Femalesara	6.072	1.190	5.559	1.024	0.462	1
Malehecale_melicerta	Femalemelpomene_rosina	7.125	1.492	6.741	0.399	0.351	1
Femaleismenius_boulleti	Malesara	7.464	0.099	6.072	1.190	1.278	1
Femalehecale_melicerta	Malemelpomene_malleti	5.815	0.105	5.398	0.588	0.769	1
Femalecydno	Malemelpomene_rosina	5.830	1.923	6.032	0.655	-0.100	1
Femalecydno	Malesara	5.830	1.923	6.072	1.190	-0.135	1
Femalecydno	Malehecale_melicerta	5.830	1.923	7.125	1.492	-0.655	1
Malemelpomene_malleti	Femalesara	5.398	0.588	5.559	1.024	-0.194	1
Femaleismenius_boulleti	Femalesara	7.464	0.099	5.559	1.024	2.030	1
Maledoris	Femalehecale_melicerta	6.799	0.970	5.815	0.105	1.009	1
Femalecydno	Femalehecale_melicerta	5.830	1.923	5.815	0.105	0.008	1
Femaleismenius_boulleti	Femalemelpomene_rosina	7.464	0.099	6.741	0.399	1.942	1
Maledoris	Malehecale_melicerta	6.799	0.970	7.125	1.492	-0.211	1
Malesara	Femalemelpomene_malleti	6.072	1.190	7.411	0.644	-1.399	1
Femalecydno	Maleismenius_boulleti	5.830	1.923	5.933	1.177	-0.046	1
Femalehecale_melicerta	Femalemelpomene_malleti	5.815	0.105	7.411	0.644	-2.692	1
Femalemelpomene_malleti	Malecydno	7.411	0.644	5.411	0.092	3.380	1
Femalehecale_melicerta	Malecydno	5.815	0.105	5.411	0.092	2.896	1
Femalesara	Malecydno	5.559	1.024	5.411	0.092	0.158	1
Malehecale_melicerta	Malecydno	7.125	1.492	5.411	0.092	1.257	1
Malehecale_melicerta	Femalemelpomene_malleti	7.125	1.492	7.411	0.644	-0.249	1
Maledoris	Malecydno	6.799	0.970	5.411	0.092	1.424	1
Malesara	Femalemelpomene_rosina	6.072	1.190	6.741	0.399	-0.753	1
Femaledoris	Malecydno	7.224	2.240	5.411	0.092	0.809	1
Maleismenius_boulleti	Malesara	5.933	1.177	6.072	1.190	-0.099	1
Femaledoris	Femalemelpomene_malleti	7.224	2.240	7.411	0.644	-0.105	1
Femaleismenius_boulleti	Femalemelpomene_malleti	7.464	0.099	7.411	0.644	0.090	1
Maledoris	Maleismenius_boulleti	6.799	0.970	5.933	1.177	0.568	1
Maleismenius_boulleti	Femalehecale_melicerta	5.933	1.177	5.815	0.105	0.101	1
Femalecydno	Femalemelpomene_rosina	5.830	1.923	6.741	0.399	-0.616	1
Femalecydno	Femalesara	5.830	1.923	5.559	1.024	0.158	1
Maleismenius_boulleti	Femalesara	5.933	1.177	5.559	1.024	0.292	1
Femaledoris	Femaleismenius_boulleti	7.224	2.240	7.464	0.099	-0.107	1

Femalehecale_melicerta	Malehecale_melicerta	5.815	0.105	7.125	1.492	-0.961	1
Femaledoris	Maledoris	7.224	2.240	6.799	0.970	0.174	1
Malesara	Femalehecale_melicerta	6.072	1.190	5.815	0.105	0.237	1
Femalemelpomene_rosina	Femalemelpomene_malleti	6.741	0.399	7.411	0.644	-1.250	1
Femalemelpomene_rosina	Malecydno	6.741	0.399	5.411	0.092	3.585	1
Femalesara	Femalemelpomene_malleti	5.559	1.024	7.411	0.644	-2.164	1
Maleismenius_boulleti	Femaleismenius_boulleti	5.933	1.177	7.464	0.099	-1.296	1
