# Supplementary figures and images for: Conserved microbiota among young Heliconius butterfly species
Source: PeerJ. 2018 Oct 2;6:e5502. doi: 10.7717/peerj.5502 (PMC6173163; doi:10.7717/peerj.5502)

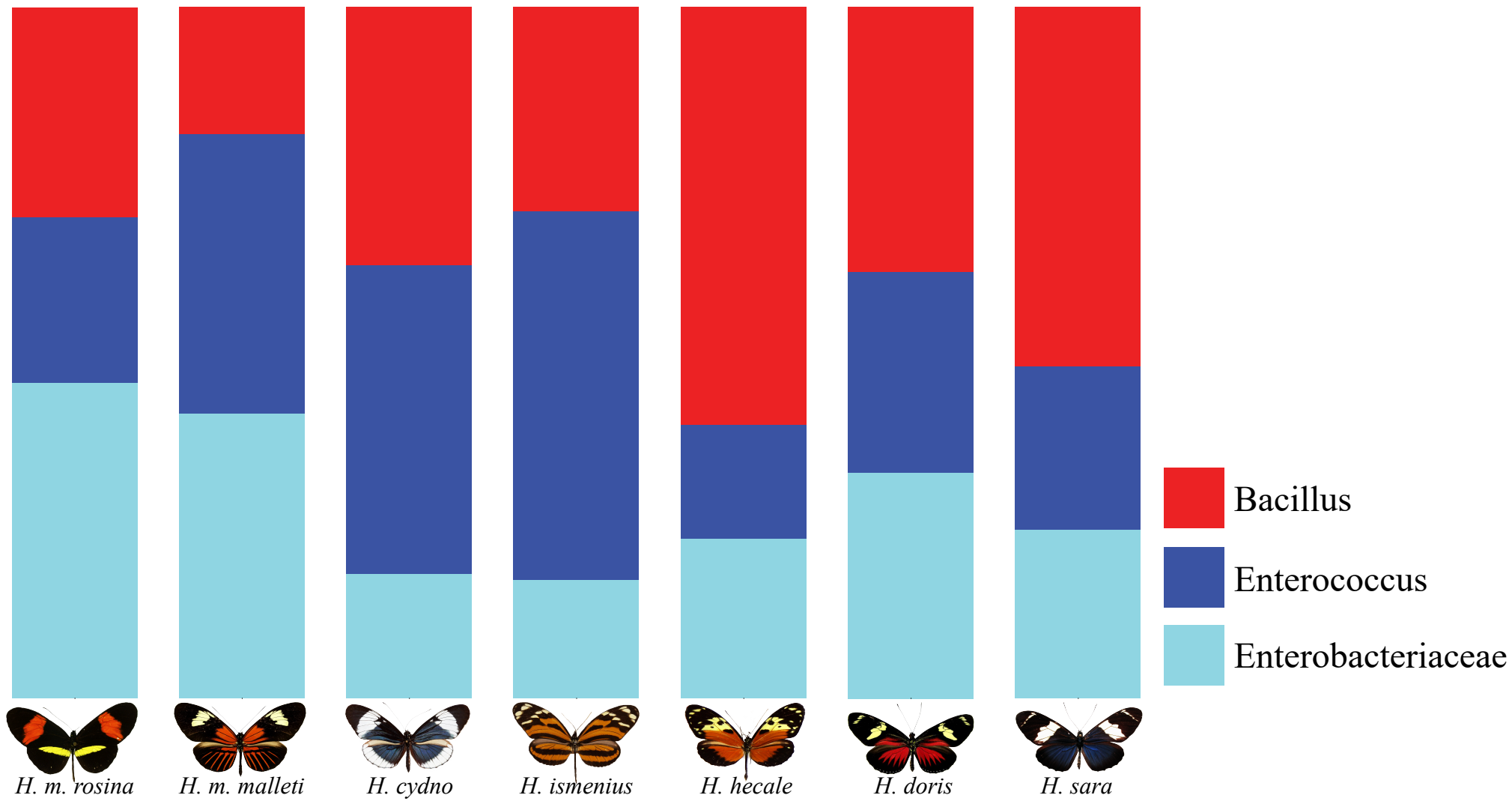

Supplement: Figure S1 — Shown is their relative abundance to each other averaged over the different (sub)species. [file peerj-06-5502-s004.pdf]

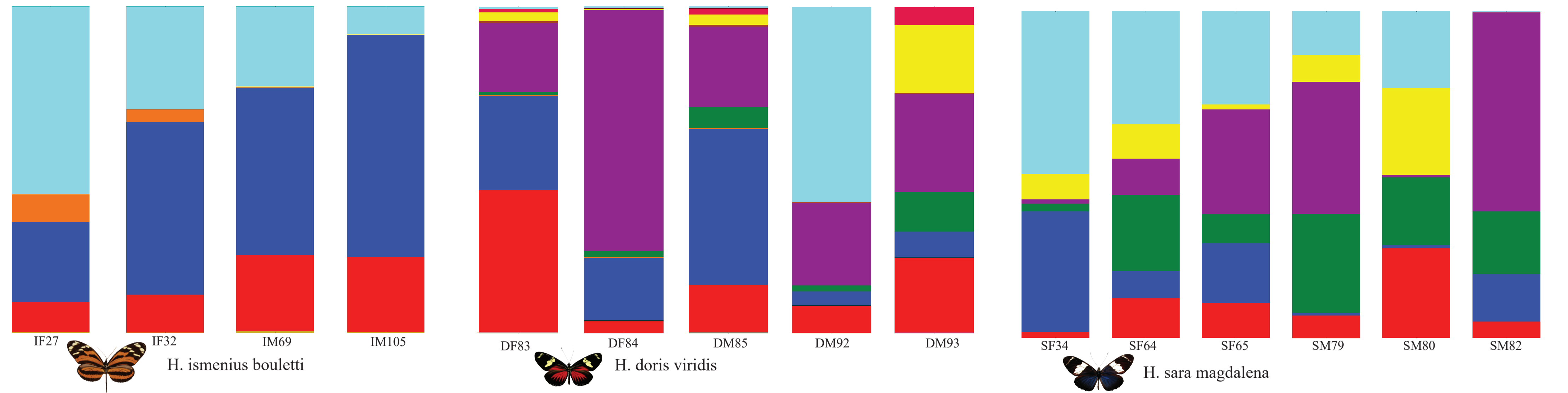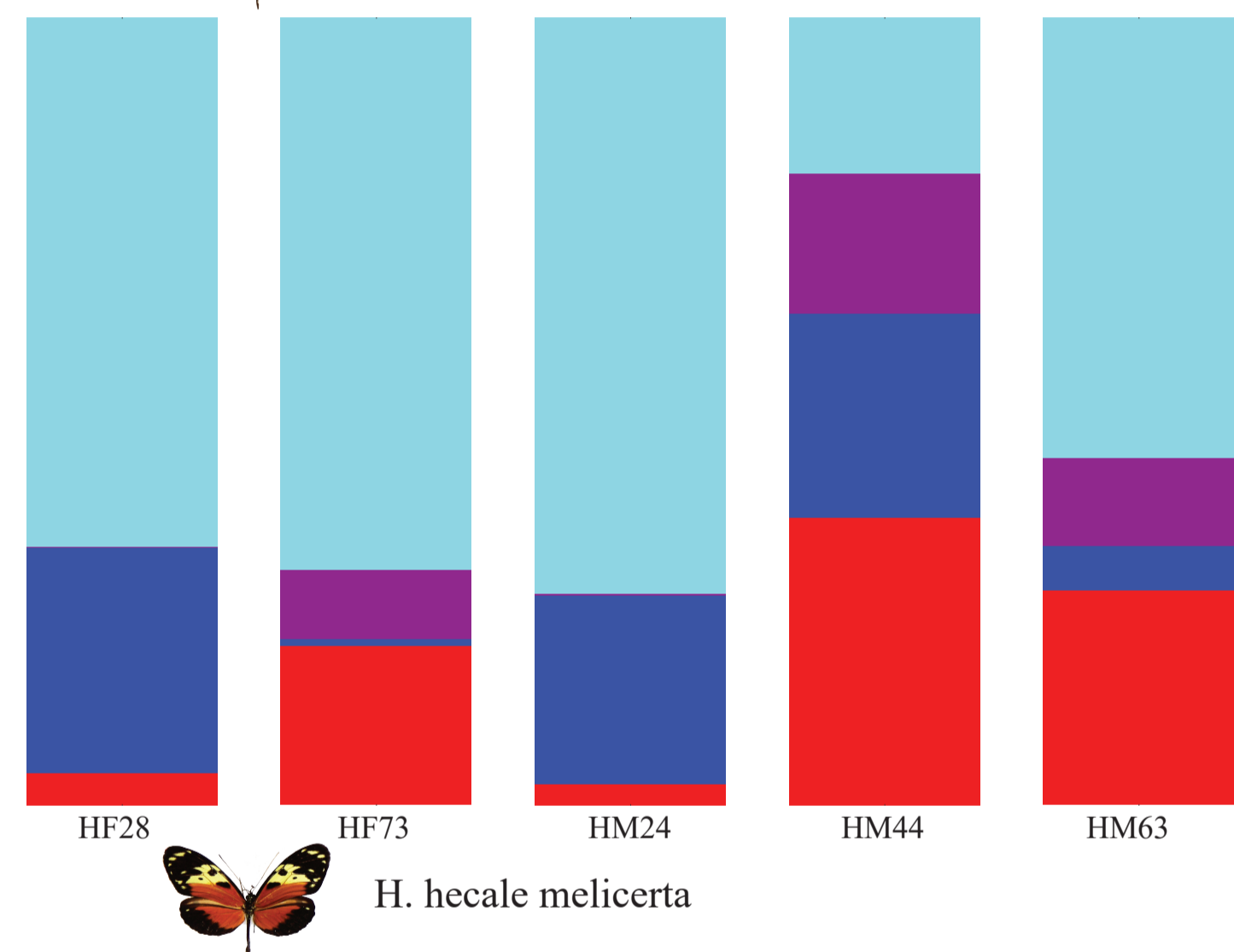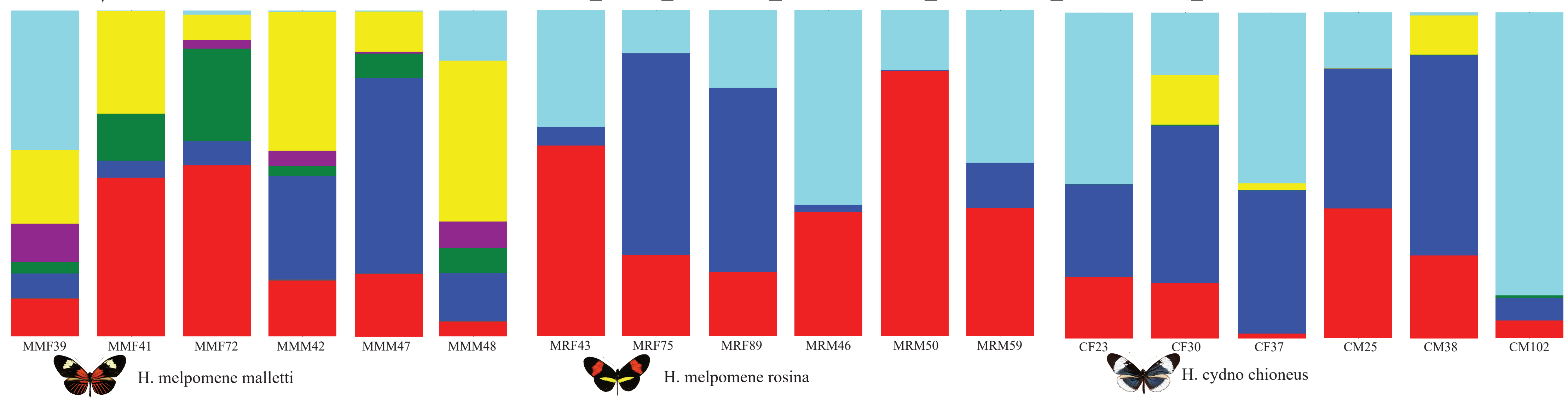

Supplement: Figure S2 — Shown is their relative abundance to each other per butterfly. [file peerj-06-5502-s005.pdf]

*Pseudonocardia*

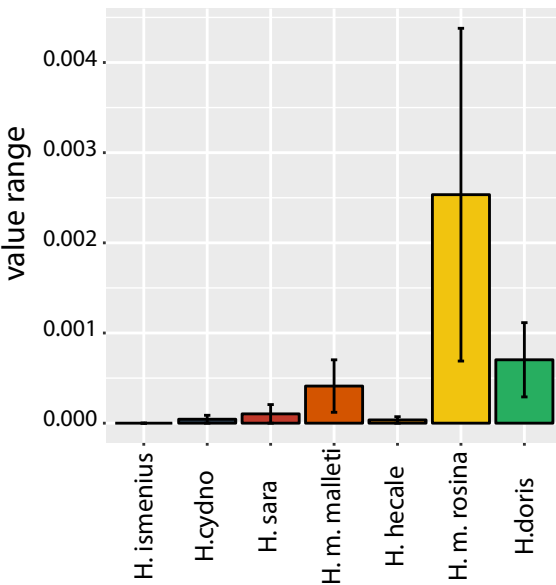

*Lactobacillales*

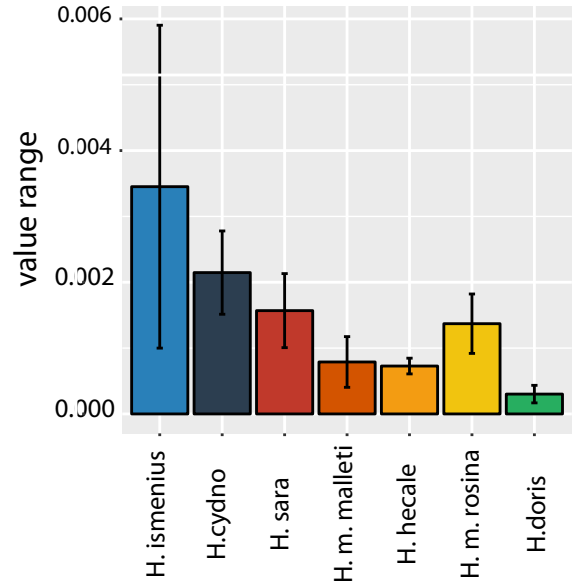

*Agrobacterium*

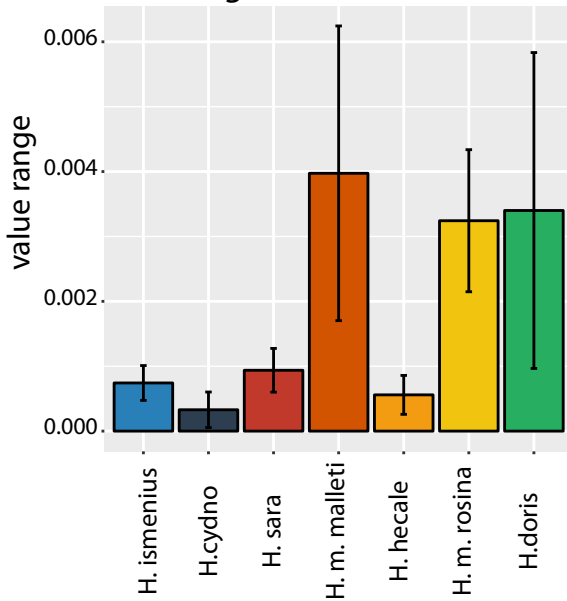

*Gluconobacter*

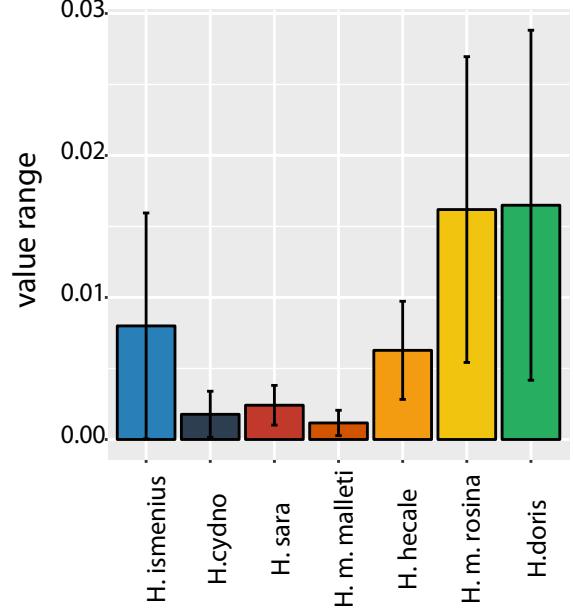

Supplement: Figure S3 [file peerj-06-5502-s006.pdf]
